# Topological Aspects of the Design of Nanocarriers for Therapeutic Peptides and Proteins

**DOI:** 10.3390/pharmaceutics11020091

**Published:** 2019-02-21

**Authors:** Nadezhda Knauer, Ekaterina Pashkina, Evgeny Apartsin

**Affiliations:** 1 Research Institute of Fundamental and Clinical Immunology, 14, Yadrinthevskaya str., 630099 Novosibirsk, Russia; nuknauer@niikim.ru (N.K.); pashkina.e.a@yandex.ru (E.P.); 2 Institute of Chemical Biology and Fundamental Medicine SB RAS, 8, Lavrentiev ave., 630090 Novosibirsk, Russia; 3 Department of Natural Sciences, Novosibirsk State University, 2, Pirogov str., 630090 Novosibirsk, Russia

**Keywords:** proteins, peptides, nanomedicine, carriers, formulations, cavitands, liposomes, dendrimers

## Abstract

Supramolecular chemistry holds great potential for the design of versatile and safe carriers for therapeutic proteins and peptides. Nanocarriers can be designed to meet specific criteria for given application (exact drug, administration route, target tissue, etc.). However, alterations in the topology of formulation components can drastically change their activity. This is why the supramolecular topology of therapeutic nanoconstructions has to be considered. Herein, we discuss several topological groups used for the design of nanoformulations for peptide and protein delivery: modification of polypeptide chains by host-guest interactions; packaging of proteins and peptides into liposomes; complexation and conjugation with dendrimers. Each topological type has its own advantages and disadvantages, so careful design of nanoformulations is needed. Ideally, each case where nanomedicine is needed requires a therapeutic construction specially created for that taking into account features of the administration route, target tissue, or organ, properties of a drug, its bioavailability, etc. The wide number of studies in the field of protein delivery by supramolecular and nanocarriers for proteins and peptides evidence their increasing potential for different aspects of the innovative medicine. Although significant progress has been achieved in the field, there are several remaining challenges to be overcome in future.

## 1. Introduction

The development of novel drug carriers is a central aim of nanomedicine. The use of carriers permits to control drug clearance, to protect a cargo from biodegradation, to achieve efficient targeting towards organs and tissues, to decrease cytotoxicity and so on [1,2]. Carriers are especially needed when therapeutics to be delivered require specific handling. 

Therapeutic peptides and proteins are very characteristic example of therapeutics of such kind. As a rule, they have short lifetime in vivo due to the fast digestion by peptidases. The typical peptide lifetime in the blood stream is several minutes [3], with the lifetime being generally shorter for non-glycosylated peptides and proteins having molecular weight less than 50 kDa. Furthermore, the majority of protein drugs cannot be administered orally due to their digestion by the proteases of the gastrointestinal tract. In respect of this, multiple injections of a drug are necessary. Such a therapy scheme is uncomfortable for patients and may result in the decrease of their compliance and potentially holds a risk of unwanted issues [1].

Thus, the development of novel methods the administration of peptide and protein therapeutics is emerged. This is a complex task that includes the search for optimal administration ways, chemical modification of amino acids to increase stability of molecules towards protease digestion, enhancement of their bioavailability. The use of specific carriers for peptide drugs (nanoparticles, liposomes, polymers, etc.) helps to solve the abovementioned task [4,5,6,7]. 

Supramolecular chemistry holds a great potential for the design of versatile and safe carriers for therapeutic proteins and peptides. Interactions at the intermolecular level permit to create tunable assemblies with controllable properties, physico-chemical, and physiological behavior. An important feature of engineered nanocarriers is the possibility to design specific constructions for given application (exact drug, administration route, target tissue, etc.). Indeed, a proper packing of peptides and proteins permits to shield them, entirely or in part, from the recognition by unwanted molecules (enzymes, off-target receptors). Packed drugs have different bioavailability and biodistribution profiles, to say nothing of an efficiency of the pharmacological action. Engineering of peptide and protein carriers opens an avenue to novel therapeutic opportunities [8,9,10,11,12,13,14].

However, the design itself is not as simple as it seems. There are many factors influencing the behavior and efficiency of peptide formulations. Among others comprehensively discussed in the references above, there exists a factor affecting the performance of nanoformulations, namely the topology of potential therapeutic constructions. Supramolecular assemblies for drug delivery are spectacularly diverse, and the spatial organization obviously plays a significant role in their activity. Furthermore, it has been shown that alterations in the intramolecular topology of macromolecules can drastically change their activity [15]. That means that supramolecular topology of nanoconstructions also has to be considered when novel therapeutic constructions are designed. 

Herein, we discuss several topological groups used for the design of nanoformulations for peptide and protein delivery: modification of polypeptide chains by host-guest interactions with cavitands (the use of cyclodextrins and cucurbiturils is discussed); packaging of proteins and peptides into liposomes; and complexation and conjugation with polymers (dendrimers are taken as a characteristic and therapeutically promising example). These examples illustrate the most common regularities of the organization of therapeutic constructions based on proteins and peptides, their advantages and disadvantages, as well as clinical relevance. 

## 2. Host-Guest Complexes of Peptides and Proteins with Cavitands

**Figure 1:**
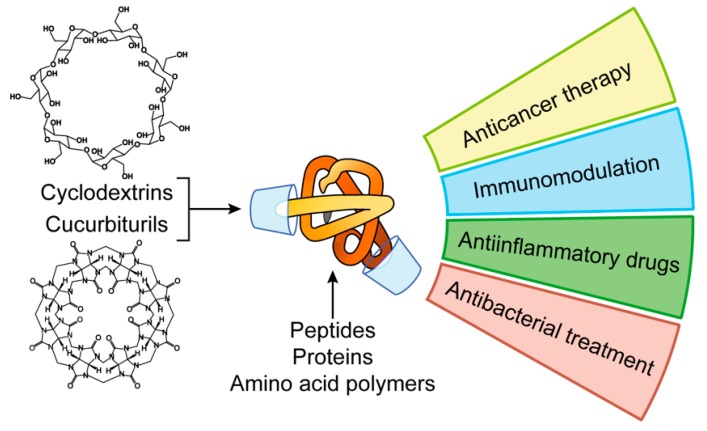
Applications of peptide and protein nanoformulations obtained by host-guest interactions with cavitands.

A way to increase the chemical stability of peptide and protein formulations in the blood stream is to protect their fragments from peptidases by including into cavitands. Cavitands are synthetic macromolecules able to form supramolecular complexes with guest molecules. Due to their chemical structure, cavitands selectively bind certain amino acid radicals in the polypeptide chain blocking them from the peptidase recognition thus increasing the stability of a protein. Herein we illustrate the role of cavitands in the peptide nanoformulation design using cyclodextrins and cucurbiturils as examples (Figure 1).

### 2.1. Cyclodextrins in Peptide Nanoformulations

Cyclodextrins (CDs) are cyclic oligosaccharides built of glucopyranose [16]. The most common CD species consist of six (α-CD), seven (β-CD), or eight (γ-CD) sugar units. CDs have hydrophobic interior and hydrophilic non-charged portals; that is why they efficiently form complexes with hydrophobic guest molecules [17]. CDs forms inclusion complexes with amino acids, peptides, and proteins. It has been shown that the interaction occurs mainly by inclusion of hydrophobic amino acid radicals into the CD cavity [18,19].

Thanks to this feature, CDs can be used in industry, including pharmacy, as stabilizers for protein formulations, such as somatotropin and insulin [20,21,22,23]. Furthermore, CDs decreases the immunogenicity of formulations, can modulate their functions, and partially protect active compounds from protease/peptidase digestion in the digestive tract upon oral administration [24].

CDs are highly biocompatible when administered in common ways [25]. Chemical modifications of the CD exterior further increase biocompatibility [26,27] by suppressing their side activities. For example, non-modified β-CD exhibits kidney toxicity upon systemic administration in vivo because of the accumulation of its complex with cholesterol as insoluble crystals [28].

Hydroxypropyl-β-CD has been shown to increase the bioavailability of calcitonin, glucagon, insulin, and recombinant human granulocyte colony stimulating factor [29]. In rats and rabbits, modified CD increased the absorption of calcitonin [30]. In rabbit model, nasal spray of liquid and powder formulation of CD-complexed glucagon greatly increased the bioavailability of the protein in comparison with the subcutaneous injection [31]. The use of low doses of CD (3–5%) increased the absorption of insulin in rats to 80–100% upon intranasal administration [32,33]. 

The use of cavitands improves the local tolerability to peptide formulations that is important upon ocular, transdermal and nasal administration [34]. As an example, the use of cyclosporine A, a hydrophobic cyclic polypeptide, to treat bacterial infections of the eye is complicated due to its poor water solubility. The use of oil solution or surfactant-containing water solutions can lead to the visual blurring or sandpaper effect in the eyes. The use of CD increases the solubility of the polypeptide and helps to avoid the side effects [35].

### 2.2. Cucurbiturils in Peptide Nanoformulations

Another type of cavitand complexing proteins and peptides is represented by cucurbiturils (CB[n]) [36]. CBs are macrocyclic co-polymers of formaldehyde and glycoluryl formed from urea and glyoxal. As CDs, CBs also have hydrophobic cavity; the portals contain highly polarized carbonyl groups [37]. Such an amphiphilic structure affects the inclusion of guest molecules: hydrophobic domains are retained in the cavity, whereas hydrophilic parts (preferentially, cationic) are bound in the portal area [38]. In general, CBs have higher affinity to cationic guests than CDs [39,40]. CB complexes with low-molecular compounds cross the cell membrane delivering a cargo into a cell [41].

An important advantage of CBs for biomedical applications is their low toxicity, even in high doses [42,43]. In particular, CB[7] is not toxic up to 1 mM in vitro and 250 mg/kg in vivo (murine model) upon intravenous injection and 600 mg/kg upon oral administration [44]. Below these doses, no systemic toxicity has been found. In very high doses, miotoxicity and neurotoxicity manifestations have been reported [45,46].

CB complexes with amino acids and their derivatives are known [47,48,49]. While complexing with therapeutic proteins and peptides, CBs form inclusion complexes with amino acid radicals in polypeptide chain, preferably, with hydrophobic aromatic side chains of phenylalanine, tyrosine, tryptophan. In the portal region, CBs bind cationic amino acid side chains of arginine and lysine [50]. CBs possess certain binding selectivity towards amino acid motifs. When bound with the series of tripeptides bearing one aromatic amino acid in any position, CB[8] bound only YGG (tyrosine-glycine-glycine) and FGG (phenylalanine-glycine-glycine) peptides that evidences the position selectivity of binding [51,52]. CBs are non-chiral, however, they discriminate l- and dl-peptides [53]. It is important taking into account that peptide therapeutics may contain d-amino acids [54].

Having bound to proteins, CBs protect them from proteases, with the protective effect being higher towards proteases recognizing cationic amino acids (e.g., trypsin) [50]. PEGylation of CBs improves the protective activity, as it has been demonstrated using insulin, glucagon and antibodies as model proteins. PEG-CB conjugate binds the polypeptides in the proximity to N-terminus blocking their interaction with exopeptidases (for example, leucinaminopeptidase) [55].

Along with the protection from peptidase digestion, CBs modulate biological properties of peptide and protein drugs. In particular, complex of CB[7] with immunomodulating peptide tuftsin has been shown to affect the cytokine-producing activity of peripheral blood mononuclear cells stimulating phagocytosis upon prolonged cultivating and increasing the antibody production in vivo [56]. The formation of inclusion complexes with CB[7] provides antimicrobial activity to polylysine [57]. CB[8] has similar effect on the phenylalanyl-poly(ethylene imine) conjugate. Interestingly, the subsequent treatment with the antibiotic amantadine provokes guest exchange in the CB[8] cavity thus eliminating the excess of an antibiotic [58].

CB[8] can include two amino acid moieties into its cavity, and that provides useful insights for bioengineering as a driving force for dimerization. For instance, caspase-9 naturally occurs mainly in the non-functional monomeric form and becomes functional only after dimerization induces by helper factors. Dang et al. introduced FGG motifs onto the N-terminus of caspase-9. Interaction with CB[8] leads to the inclusion of two phenylalanine moieties from different protein molecules into the cavity that caused the dimerization of the enzyme into its functional form [59].

In summary, site-specific decoration of peptides and proteins via host-guest inclusion into cavitands represents an easy and efficient way to modulate physico-chemical properties of therapeutic protein formulations. Through that, the pharmacological behavior of therapeutics (binding with serum proteins, circulation time, biodistribution, targeting) is also changed. Although not yet well developed, this approach holds great potential for the design of novel agents for nanomedicine.

## 3. Liposome-Encapsulated Proteins and Peptides

**Figure 2:**
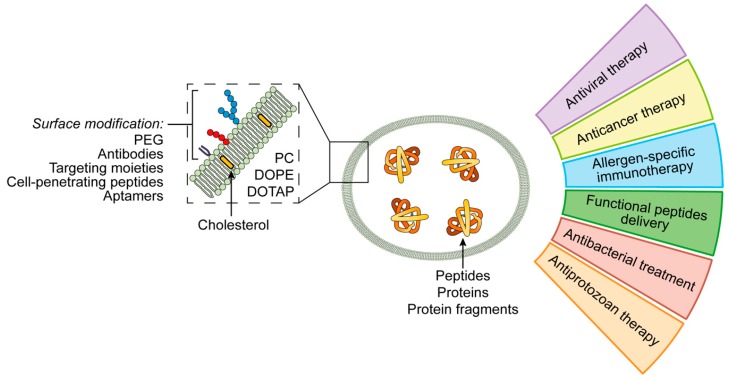
Applications of liposomal peptide and protein nanoformulations.

Liposomes are closed vesicle nanoparticles formed of bilayers of amphiphilic molecules. Due to the high permeability, low toxicity, biocompatibility and structural flexibility, they are widely used as carriers for drug delivery [60,61]. The most common building blocks of liposomes are natural phospholipids such as phosphatidylserine, phosphatidylcholine, phosphatidylethanolamine, and their derivatives [62,63] (Figure 2).

Liposomes can carry both low- and high-molecular cargo, either lipophilic one integrated in their membrane or hydrophilic one entrapped in the aqueous interior [61,62,64]. Amphiphilic molecules such as proteins and peptides are the optimal cargo of choice [65,66].

### 3.1. Features of the Liposome Design for Peptide Delivery

In the blood stream, non-modified liposomes are quickly opsonized and uptaken by phagocytes. After the binding with opsonins, a part of liposomes undergoes degradation. The degradation process is affected by the size and surface charge of a particle: large non-modified liposomes are eliminated faster than small neutral or cationic liposomes [64].

To improve the performance of liposomal system for systemic drug delivery, peculiarities of lipid nanoparticles’ interactions with blood and tissues should be taken into account. An important point is how to safely increase the circulation time of liposomes. In particular, PEGylation of liposomes (decoration of the liposome surface with polyethylene glycol, PEG) prevents their binding with serum proteins and reduces their uptake by reticuloendothelial system (RES) [67]. The protective effect of PEG depends on its molecular weight [61,64,68]. However, PEGylation has unwanted side effects (such as decrease of the affinity of liposomes to a cell membrane and hindered drug release in cytosol) that should be also taken into account [61]. An important feature of liposomes is the possibility to introduce targeting moieties onto the surface of therapeutic constructions. It can be made using directing site-specific ligands: antibodies to the cell surface ligands, cell-penetrating and cell-targeting peptides, aptamers, small molecules (affibodies), or their combinations [60,67,69]. As many other drug delivery systems penetrating into a cell by endocytosis, following the internalization, liposomes face the issue of the endosomal escape. This is especially important if the cargo is a peptide that is digested during the maturation of an endosome [61]. This problem can be solved by using the stimuli-sensitive liposomal systems which release the therapeutic in special conditions (pH, temperature, ultrasound, or magnetic field) [60,70].

It should be noted that liposomes are not entirely biologically inert, and the functional groups on their surface can stimulate the immune response [62]. The decreased of the circulation time in the blood stream was described after the repeated administration of PEGylated liposomes. This fact called ABC-phenomenon (accelerated blood clearance) represents an important obstacle for the using of therapy protocols with repeating liposomal constructions administration [64]. The mechanism of the ABC is still unclear; it is affected by such factors as amount of lipids injected, PEG coverage degree, interval between doses. The repeated injections of liposomal drugs to rats lead to the decrease of the circulation period and increase the level of anti-PEG IgM. This effect was not observed after a splenectomy. The use of higher doses of liposomes induces the decrease of the number of PEG-specific B cells and anti-PEG IgM production [64]. Another important point of concern is the activation of the complement system and pseudoallergic reactions (so-called complement activation–related pseudoallergy) leading to the facial swelling, headache and sometimes anaphylaxis [67]. The promoting factors are liposome heterogeneity, surface charge, the large size of the particles or presence of the liposomal aggregates, high cholesterol content, and PEGylation [61,64].

Interestingly, the components of liposomes affect the type of immune response. Treating mice with nanoemulsions of soybean phospholipids promotes the activation and maturation of dendritic cells increasing the level of Th1 cytokines [71]. Vaccines containing *Leishmania major* surface glycoprotein rgp63 with distearoylphosphatidylcholin (DSPC) stimulate Th1 immune response in mice models [72].

Moreover, such factors as lipids fluidity or surface charge can also play a crucial role in the immune response modulation. Solid liposomes containing lipids with high phase transition temperature induce more intense immune response towards membrane antigens. Fluid lipid-derived liposomes are preferred for antigens that require to be processed by antigen-presenting cells (APCs). The rigidity of membranes prevents the intracellular antigen release but improves the interaction of the APCs cell membrane with that of the affecting lymphocytes [73]. For example, the use of dioleylphosphatidylethanolamine (DOPE) results in releasing of a peptide drug to the cytosol. That leads to the presentation of a peptide on the cell surface in the complex with the MHC I molecules where it can be identified by CD8+ cytotoxic lymphocytes [68]. Cationic liposomes demonstrate better uptake by the APCs than anionic or neutral ones [73].

Liposomes are flexible and highly tunable nanocarriers of peptide and protein drugs for various applications. Given below are examples of the use of liposomal peptide/protein formulation in nanomedicine (Figure 2).

### 3.2. Treatment of Infectious Diseases

Liposomes can be used to transport antigens inducing immune response, with lipid component having its proper activity as an adjuvant. As an example, an HBsAg-containing vaccine based on liposomes of soybean phospholipids has been reported; the construction induced the increase of the specific IgG production, stimulation of mucosal immunity and Th1-type polarization of the immune response [74]. Similar results have been obtained using other microorganisms as antigens (vaccinia virus, respiratory syncytial virus) [75,76]. The design of anti-HCV vaccines containing liposome-entrapped HCV epitopes was also reported. The administration of such construction stimulated cytotoxic immune response [77]. It is worth noting that packaging of protein antigens into nanocarriers can distort the epitopes’ structure or shield them that prevents the development of fully functional immune response.

Liposomes can carry peptides directly affecting causative pathogens. There are liposomal systems containing antimicrobial peptides against *E. coli, P. aeruginosa, K. pneumonia*, or anti-fungal peptide against *Aspergillus fumigatus* [63]. Liposomal formulations have better antimicrobial activity as compared with free peptides. Formulations of nisin and lysozyme suppress the development of *Listeria monocytogenes* [78]. Liposomal constructions with anti-protozoan activity have been also reported. The use of the liposomal vaccine containing the *Leishmania donovani* antigen in combination with monophosphoryl lipid A decreased the parasite titer [77]. A substance for the transdermal anti-plasmodia immunization is described. This substance consists of liposome-entrapped C-terminal fragment of the merozoite-1, the surface protein of *Plasmodium falciparum* [79].

### 3.3. Antitumor Therapy

The delivery of tumor-associated antigens-loaded liposomes induces antitumor immune response is a prospective way of the cancer therapy. Arab et al. have found interesting effects in the BALB/c mice model after the treatment by DOPE-derived constructions containing E75 peptide (HER-2/neu-369–377) which is highly expressed in patients with breast cancer. When the tumor cells were injected for the tumorogenesis stimulation, treated mice had smaller tumors and higher survival rate. If mice already had tumors, the treatment suppressed the tumor development and resulted in the prolongation of the survival period. It can be explained by the cytotoxic CD8-mediated immune response [68]. The same results have been obtained upon the administration of the construction containing monophosphoryl lipid A and P5 peptide (containing rat HER2/neu epitopes) [70].

Rueda et al. created the vaccines against prostate cancer containing artificial peptides stimulating the immune response against luteinizing-hormone-releasing hormone (LHRH). They used the complex of LHRH and tetanus anatoxin, TLR-ligands Pam_3_CSK_4_, R848, and polyinosilic-polycytidilic acid (poly-I:C) entrapped in the liposomes containing PC, PG, DSPE, and PEG. These constructions stimulated the immune response against the hormones and can be possibly used for the anti-hormonal therapy of the prostate cancer [80].

The combination of TLR-ligands (poly(I:C), Pam_3_CSK_4_, CpG) with synthetic peptides and cationic DOTAP-liposomes were efficient in the mice models of melanoma and HSV-associated tumors. Varypataki et al. used ovalbumin for melanoma model and HSV-derivated E7 oncoprotein for HSV-associated tumors. The stimulation of antigen-specific CD4 and CD8 immune response was demonstrated [81].

Liposomal drugs can be also used for apoptosis induction [82]. The effects of liposomes loaded with TRAIL molecules (tumor necrosis factor-related apoptosis inducing ligand) and actinomycin D-loaded liposomes on the tumor cells line A549 were studied in the mice model of non-small cells lung cancer. This combination provided the synergetic anti-tumor effect resulting in the suppression of the growth of the subcutaneous xenograft. Actinomycin D sensitized tumor cells to the TRAIL and apoptosis induction. The same effect has been shown upon the use of liposomes co-loaded with TRAIL and doxorubicin.

### 3.4. Treatment of Allergic Diseases

Liposomes can be used to develop formulations for the allergen-specific immunotherapy (ASIT) [83]. For instance, an intraperitoneal injection of liposomes loaded with *Artemisia scoparia* extract to the sensibilized mice lead to the Th1 polarization and induction of regulatory T cells (Tregs) proliferation [84]. Subcutaneous administration of the liposomal dust mite allergens for the patients with bronchial asthma resulted in the decrease of the level of the specific IgE, as well as in the clinical improvement [85].

Nevertheless, despite of the liposome allergen formulations being efficient in the injectable form, it seems prospective to use them as mucosal vaccines stimulating local and systemic immune response. This allows to escape of the risks of the injection and to improve the patients’ compliance. The intranasal use of *Dermatophagoides pteronyssimus* allergens entrapped in liposomes containing PC and cholesterol leads to lower IL-5 and IL-6 expression, higher expression of IL-10, IL-35, and TGFβ after the allergen provocation [86].

Treating mice with ovalbumin-loaded PC-liposomes sublingually resulted in the low number of eosinophils in the bronchoalveolar lavage and decreased level of specific IgE. Splenocytes from the treated mice proliferated less actively in comparison with control; the IL-5 and IFNγ production also decreased [87]. In the mice model of allergic rhinitis, the intranasal liposome-based vaccine with cat allergen *Fel d1* caused the clinical improvement and shifted the Th2 immune response towards Th1 and Treg responses [88]. The similar results have been also shown in the model of atopic bronchial asthma in mice sensitized to the cockroach allergens [89].

### 3.5. Delivery of Miscellaneous Peptide Drugs

Liposomes can be used for the delivery of some other types of peptides with various activities. As an example, liposomal formulations of ghrelin, including nasal ones, have been designed [90,91]. Ghrelin is the peptide hormone produced by the cells in the gastrointestinal tract which regulates appetite and body weight changings. It also reduces the level of pro-inflammatory cytokines taking part in the cachexia development. However, it undergoes fast proteolytic degradation in physiological media. Salade et al. constructed chitosan-coated liposomal system for nasal administration of ghrelin for patient with cachexia [90]. Encapculation of ghrelin into liposomes increased its biostability, whereas chitosan coating provided enhanced mucoadhesion and permetation through epithelium monolayer.

The inflammation is accompanied by oxidative stress and treating this condition by superoxide dismutase potentially results in the clinical improvement. The current data demonstrate that small PEGylated liposomes (<100 nm diameter) can be delivered into the inflammation loci without significant degradation. This formulation can be potentially used in patients with rheumatoid arthritis [92].

Functional food can also contain liposomal constructions [66]. As an example, rainbow trout skin-derived peptides encapsulated in chitosan-covered liposomes can be the perspective candidate for antioxidant compounds delivery [93].

Promising results were obtained by the group of P. Mura, who developed the liposomal form of opiorphin. This peptide was first isolated from the saliva and demonstrated the high painkilling effect without a risk of addiction. However, opiorphin molecules degrade quickly after the intravenous injection. It was demonstrated that using of PEGylated liposomes can protect the drug from plasma peptidases and extend its effect. Furthermore, liposomal opiorphin can be administered intranasally in the complex with hydrogels. This route is very comfortable for patient and may be used for the nose-to-brain delivery bypassing the blood-brain barrier [94].

The data presented suggest that liposomes are efficient carriers for the peptide and protein drugs. Further advances will be connected with new compounds to stabilize liposome formulations, with their targeting, as well as with new drugs being delivered.

## 4. Complexes and Conjugates of Proteins and Peptides with Dendrimers

**Figure 3:**
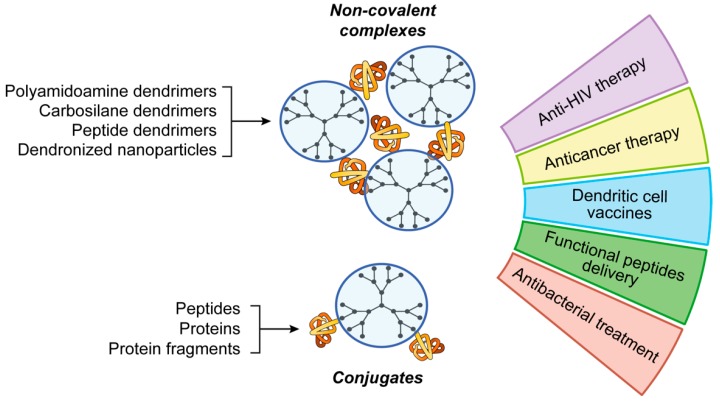
Applications of dendrimers for peptide and protein delivery.

Polymers are widely used for protein and peptide delivery [95]. Herein, we emphasize the use of one class of polymers, namely dendrimers—highly symmetric hyperbranched polymers. In the recent years, dendrimers attracted the great interest in nanomedicine as prospective carriers of biologically active molecules, including macromolecules. As “classic” polymers, dendrimers are built of repeating structure blocks. However, their molecular topology is different: dendrimers consist of the core and branches containing periodically positioned branching points. Due to the structure features, fully symmetric dendrimers are spherical molecules exposing large number of equivalent functional groups on the periphery. Such a multivalency permits many functional moieties to be conjugated with one molecule of dendrimer. Alternatively, multiple interactions of dendrimer with macromolecular cargo are possible (Figure 3).

It should be noted that despite of structural and functional similarity between dendrimers and non-symmetric polymers, the formers have an important advantage: dendrimers are monodisperse molecules possessing exact molecular formula and synthesis-programmed structure. This feature does not seem to be a self-evident advantage of dendrimers in view of increasing complexity of the synthetic procedures. However, it can facilitate the certification of dendrimers for biomedical use by governmental regulators (European/US FDA, etc.). In particular, a dendrimer-based drug Vivagel^®^ preventing the HIV infection has been approved for using in condom lubricants [96,97]. It has also been subjected to clinical trials in the bacterial vaginosis treatment [98,99]. These recent positive findings stimulate the development of new dendrimer-based constructions for future medicine.

To date, a wide number of dendrimers has been synthesized, with their properties varying depending on architecture [100]. For a long while, the use of dendrimers as carriers for therapeutically relevant macromolecules was limited to the delivery of nucleic acids (for the recent data, refer, for example, to [101,102]). Recently, dendrimers have aroused interest as delivery systems for proteins and peptides (Figure 3).

The development of drug delivery systems starts from the rational design of carriers. In the case of dendrimer-based functional materials, parameters of their design have been summarized by Tomalia [103,104,105]: particle morphology, self-organization, ability to interact with other molecules and macromolecules, etc. The concept of dendrimer space introduced by Majoral and Mignani [106,107] defines the requirements to the dendrimer architecture from the point of view of nanomedicine: bioinertness of the dendrimer scaffold (or, on the contrary, its biodegradability); presence of functional groups to bind biologically active compounds; structural features to achieve efficient internalization into a cell, extended circulation time, and induction of endosomal release. All these parameters are taken into account while constructing dendrimer-based systems for peptide and protein delivery.

Generally, peptides and proteins are delivered as complexes with cationic dendrimers (referred to as dendriplexes). In dendriplexes, a cargo is bound by electrostatic interactions and shielded by dendrimer molecules. Ionov et al. studied the complexation of three HIV-specific peptides (Gp160, P24, and Nef) with cationic carbosilane dendrimers [108]. Peptides are fully bound at the 10-fold excess of cations; the complexation is accompanied by the increase of the polarization of peptide fluorescence. The dendriplexes have stable strongly positive surface charge. It should be noted that peptides are released upon time; the release rate depends on the chemical structure of a dendrimer.

Dendriplexes penetrate into a cell presumably by endocytosis. However, the opsonization of dendriplexes does not always end by the endosome opening followed by the peptide release into the cytosol. In this unfavorable case, the endosome interior acidifies, and the compartment evolves into lysosome, where a peptide cargo is digested by proteases. In view of this, carrier constructions terminating the endosome maturation are of great interest. The termination is achieved by preventing acidification or by disrupting the endosomal membrane. Chang et al. reported a protein and peptide delivery system based on polyamidoamine (PAMAM) dendrimers [109]. Generation 4 (G4) dendrimer bearing 64 primary amino groups was modified by 4-guanidinobenzoic acid moieties (~60 moieties per molecule). Guanidyl groups in the dendrimer structure were engaged into the binding with a peptide cargo, whereas aromatic fragments were responsible for the disruption of endosomal membrane. To prove the efficiency of the system designed, the researchers delivered model peptides (cyclic undecapeptide CWMSPRHLGTC and heptapeptide AVPIAQK) and proteins (human serum albumin, R-phycoerythrin, β-galactosidase, p53 protein, and saporin) into a cell.

More complex peptide delivery system has been reported in [110]. As a platform for the carrier, polyacrylate-coated superparamagnetic Fe_3_O_4_ nanoparticles have been chosen. The surface of nanoparticles has been decorated with G3 polylysine dendrons modified with carboxybetaine (trimethylammonium acetate) moieties on the periphery. Dendron branches contained thermoresponsible elastin-like VPGVG pentapeptides between G2 and G3 branching points. Dendronized nanoparticles efficiently bound vascular endothelial growth factor (VEGF) and retained it in a complex at physiological conditions. The application of an oscillating magnetic field induced the heating of magnetic nanoparticles leading to a local hyperthermia. At the temperature >42 °C, elastin-like peptides denatured followed by the release of the VEGF.

An important application of dendrimer complexes with proteins and peptides is the delivery of antigens into antigen-presenting cells [111]. In perspective, the targeted antigen delivery will make it possible to do antigen-specific immunotherapy of various diseases including allergies, bacterial infections, cancer, and so on.

García-Vallejo et al. reported a dendrimer modification improving their penetration into dendritic cells. G0-G7 PAMAM dendrimers were functionalized with Le^b^ glycans consisting of CKOT1 (SIINFEKL) or CKOT2 (ISQAVHAAHAEINEAGR) peptide and GlcNAc(Fuc)-Gal-Fuc oligosaccharide [112]. Le^b^ glycan is a natural ligand of the receptors on the dendritic cell surface. The Le^b^ glycan multivalency (i.e., presence of several ligand moieties in one molecule) increase the dendritic cells avidity and facilitates the internalization of dendrimers. The Le^b^-modified dendrimers enhance the proliferation of antigen-specific CD4+ and CD8+ lymphocytes. They do not induce the dendritic cells maturation, however, enhance the interleukin-10 proliferation. Among the series of modified PAMAM dendrimers, G3 dendrimer possessed the most pronounced activity. Such a construction has been suggested for the targeted delivery of antigens into dendritic cells.

Moura et al. have done the computer modelling of the poly(glutamic acid) dendrimers for the targeted delivery of melanoma-associated antigens MART-1, gp100:44–59 and gp100:209–217 into antigen-presenting cells [113]. As a core, dendrimers contained nitrobenzoxadiazole, a fluorophore permitting to monitor the internalization of dendrimer into cells. G4 dendrimer and its conjugates with antigens or mannose amine moieties have been chosen for modeling. The geometry of dendrimers was optimized following by the docking with the mannose receptor on the cell surface. The docking has shown that the conjugates of gp100:44–59 and gp100:209–217 antigens with the dendrimer bearing 16 mannose amine moieties have the highest affinity to the receptor. These conjugates are potentially able to penetrate into antigen-presenting cells.

Despite the emergency of the topic and promising preliminary results, there are limited data on the biological effects of the complexes of dendrimers with proteins and peptides. Kojima et al. used dendrimers as a platform to deliver ovalbumin and amyloid-promoting peptide derived from the helix B of ovalbumin (hB peptide, ovalbumin fragment ^32^IAIMSA^37^) [114]. G4 PAMAM dendrimer was simultaneously modified with guanidine fragments (15 moieties per molecule) and hB peptide (3 moieties per molecule). Dendrimers complexed ovalbumine to form 200 nm-size dendriplexes. The presence of hB peptide in the dendrimer structure seems to increase the stability of dendriplexes and to protect ovalbumin. Complexes of ovalbumin with a dendrimer without hB moieties aggregates after three days’ storage. Modified dendrimers efficiently delivered ovalbumin and peptide into RAW264 murine macrophages. Such a delivery method has been considered to help treat egg protein allergy.

Another application of dendrimer-based peptide delivery having high clinical importance is the delivery of bacterial antigens into antigen-presenting cells to fight against bacterial infections. As an example, *Chlamydia* sp. are the leading cause of sexually transmitted bacterial infections. In particular, *Chlamydia trachomatis* causes a genital infection and is responsible for the development of trachoma leading to blindness. There is a conjugate and a non-covalent complex of G4 OH-terminated PAMAM dendrimer with the AFPQFRSATLLL peptide, a mimic of glycolipid antigen of *C. trachomatis* [115]. Subcutaneous injections of peptide-dendrimer formulations induced the synthesis of chlamydia-specific antibodies in murine models, with the efficacy of induction being comparable with that of the free peptide mixed with the standard adjuvant, aluminum oxide. The dendrimer improved the stability of the peptide. The immunization of mice with these formulations led to the decrease of the bacteria titer and smoothened the manifestation of genital infection.

The data presented herein suggest that dendrimers can be considered as prospective and highly efficient carriers of proteins and peptides into a cell. The chemical and topological flexibility of dendrimers permitting to deliver peptide antigens both as conjugates and as non-covalent complexes opens an avenue for the development of formulations for efficient and safe immunization.

It should be noted that the data reported to date concern just limited number of dendrimer types, presumably PAMAM dendrimers. In the meantime, many classes of dendrimers of various architecture (phosphorus, carbosilane, polyester, and polylysine dendrimers, to name a few) possessing different biological activities (including immunostimulation [15]) and biodistribution patterns have been synthesized and successfully used in nanomedicine. Undoubtedly, further advances in this field will lead to novel formulations for peptide and protein-based therapy.

## 5. Discussion

**Figure 4:**
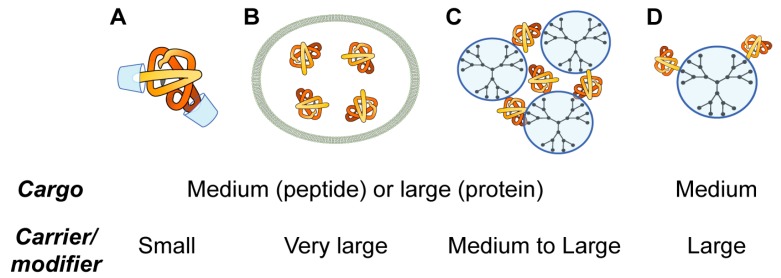
Topological groups of nanoformulations for peptide and protein delivery: modification of polypeptide chains by host-guest interactions with cavitands (**A**); packaging of proteins and peptides into liposomes (**B**); complexation with dendrimers (**C**); and conjugation with dendrimers (**D**).

Herein, four topological types of the organization of nanocarriers of therapeutic proteins and peptides (host-guest interaction with cavitands, liposomal entrapment, complexation and conjugation with polymers) are discussed. The choice of these types is not aimed to be comprehensive but to give the insight to the typical topologies used in supramolecular chemistry for the design of nanomedicines: medium or large cargo and small carrier/modifier; medium or large cargo and very large carrier (bilayer); medium or large cargo and large carrier (Figure 4). Each type has its own advantages and disadvantages (Table 1), so careful design of nanoformulations is needed. Ideally, each case where nanomedicine is needed requires a therapeutic construction specially created for that taking into account features of the administration route, target tissue or organ, properties of a drug, its bioavailability, and so forth.

Along with the use of individual topologies, they can be combined in one construction to compensate drawbacks and acquire advantages. To date, several types of combinations of the abovementioned topologies have been attempted. The combination of host-guest inclusion and liposome entrapment permits to increase the solubility of a cargo, to stabilize formulations and decrease their toxicity, as well as to achieve controllable release of a cargo from the complex with a carrier. An unwanted event of short-term sharp increase of the drug concentration upon the liposome disruption is thus avoided [116,117]. There are examples of such combination including cyclodextrins [118], cucurbiturils [119], and calixarenes [120,121]. Dendrimers have also been combined with cavitands to increase the therapeutic performance of drugs [122]. There are examples of the use of cyclodextrins [123] and cucurbiturils [124] together with dendrimers to deliver amino acids and peptides into cells.

These data suggest that the design of multi-component and multifunctional systems for the delivery of therapeutic peptides and proteins is an interesting branch of nanomedicine that is now in the beginning of development. There are prospective growing points that can lead to highly efficient innovative therapeutics. Undoubtedly, future research will contribute to the field advancing it towards clinical use.

## 6. Conclusions

The wide number of studies in the field of protein delivery by supramolecular and nanocarriers discussed here and in other reviews evidence their increasing potential for different aspects of the innovative medicine. Although significant progress has been achieved in the design of novel nanoformulations for the delivery of proteins and peptides, there are several remaining challenges to be overcome in future. For instance, the design of safe, stable, and efficient peptide-containing therapeutic nanoconstructions that can be subjected to clinical trials is still of great importance. In view of the progress made and the latest advances in the supramolecular chemistry, we believe that this task can be solved. Further advances in the carrier targeting are also required. If properly designed and assembled, peptide nanoformulations would become valuable tools for biomedical uses, such as antitumor therapy, vaccination, allergy treatment, and so on. Moreover, it is very likely that the development of the peptide and protein nanocarriers will lead to better understanding and following modulation of their properties as key components of sophisticated therapeutic systems.

## Figures and Tables

**Table 1:** Advantages and disadvantages of the topological types discussed.

Topological Type	Advantages	Disadvantages
Host-guest interaction	- Protection of peptides from proteases; - Modulation of pharmacological properties; - High stability of complexes upon storage.	- Binding with limited number of moieties or motifs; - Release rate cannot be controlled; - Safety studies required.
Liposomal entrapment	- High biocompatibility of formulations; - Flexible architecture and composition: tunable formulations possible; - Good protection of a cargo.	- Low stability of nanoassemblies’ dispersions; - High polydispersity of formed nanoconstructions.
Complexation with dendrimers	- High loading capacity; - Flexible composition of formulations; - Good protection of a cargo.	- Polydispersity of carrier molecules (polymers)/multi-step synthesis (dendrimers); - Polydispersity of formed nanoconstructions; - Safety studies required.
Conjugation with dendrimers	- Chemically controllable structure cargo loading; - Physico-chemical stability of formulations; - Easier certification for clinical use.	- Cargo release should be synthetically allowed; - Safety studies required.
